# ALYREF-mediated RNA 5-Methylcytosine modification Promotes Hepatocellular Carcinoma Progression Via Stabilizing EGFR mRNA and pSTAT3 activation

**DOI:** 10.7150/ijbs.82316

**Published:** 2024-01-01

**Authors:** Jiayida Nulali, Kaiwen Zhang, Manmei Long, Yueyue Wan, Yu Liu, Qianyue Zhang, Liu Yang, Jun Hao, Linhua Yang, Huaidong Song

**Affiliations:** 1 The Core Laboratory in Medical Center of Clinical Research, Department of Molecular Diagnostics and Endocrinology, Shanghai Ninth People's Hospital, Shanghai Jiao Tong University School of Medicine, Shanghai, 200011, China.; 2 Department of Pathology, Shanghai Ninth People's Hospital, School of Medicine, Shanghai Jiao Tong University, Shanghai 200127, China.; 3 Department of Respiration, Yangpu Hospital, Tongji University School of Medicine, Shanghai, China.; 4 Department of Liver Surgery, Renji Hospital, School of Medicine, Shanghai Jiao Tong University, Shanghai 200127, China.; 5 Department of Biliary-Pancreatic Surgery, Renji Hospital, School of Medicine, Shanghai Jiao Tong University, Shanghai 200127, China.

**Keywords:** M5-methyladenosine (m5C), Liver hepatocellular carcinoma (LIHC), Aly/REF export factor (ALYREF), EGFR, Epithelial-mesenchymal transition (EMT)

## Abstract

5-Methylcytosine (m5C) is one of the most ubiquitous modifications of mRNA and contributes to cancer pathogenesis. Aly/REF export factor (ALYREF), an m5C reader, is associated with the prognosis of liver hepatocellular carcinoma (LIHC). However, the effects of ALYREF on the progression of LIHC and the underlying molecular mechanisms remains elusive. Through an analysis of an online database and 3 independent LIHC cohorts, we found that ALYREF was markedly elevated in human liver cancer tissues and was significantly correlated with LIHC clinicopathological parameters, including Ki67^+^ cell rate, high-grade TNM stage, and poor prognosis. Several experiments were conducted to investigate the molecular basis and functional role of ALYREF-related progression in this study. ALYREF could enhance LIHC cell proliferation, migration, invasion, and epithelial-mesenchymal transition (EMT) *in vitro* and tumor formation *in vivo*. Mechanistically, ALYREF promoted the progression of human LIHC through EGFR pathways. Furthermore, ALYREF could directly bind to the m5C modification site of EGFR 3' untranslated region (3' UTR) to stabilize EGFR mRNA. Collectively, ALYREF played a crucial oncogenic role in LIHC via the stabilization of EGFR mRNA and subsequent activation of the STAT3 signaling pathway. Our results may help to elucidate the potential mechanisms of ALYREF-induced m5C modification in the progression of human LIHC.

## Introduction

Liver hepatocellular carcinoma (LIHC) is one of the most common malignant tumors in the world, with high morbidity and recurrence rates 1. Although great advances have been made in clinical diagnosis and cancer treatment, the overall prognosis of LIHC is still poor due to the high frequency of metastases and tumor recurrence 2. Therefore, it is critical to elucidate the molecular mechanisms of LIHC initiation and progression.

Among the hundreds of chemical modifications identified, 5-methylcytosine (m5C) is a highly focused epigenetic modification that has been identified in different RNA families 3. Accumulating evidence has demonstrated that m5C modification regulates many biological processes of RNA, such as RNA translation 4, 5, RNA stability 6, 7, and RNA export 8. As an m5C 'Reader' (modified RNA binding proteins), the export factor Aly/REF (ALYREF) can recognize and bind to RNA m5C methylation sites, which influence the splicing, stability, or gene transcription of mRNA 9-11. ALYREF was dysregulated in human cancers and was linked to poor survival 12-15. Recent studies have indicated that ALYREF may function as a prognostic factor and is related to immune cell infiltration in LIHC 16, 17. Although ALYREF is an important component of m5C-related genes, the detailed molecular function of ALYREF in LIHC and its underlying mechanism have not been fully elucidated.

EGFR is a transmembrane receptor tyrosine kinase protein that belongs to the ErbB family and is considered dysregulated in many different types of tumors 18-21. Therefore, therapies targeting EGFR have been developed to treat patients with LIHC 22. Increasing evidence has confirmed that EGFR is important for the development of tumorigenesis in LIHC 23. Other studies have indicated that EGFR contributes to its constitutive activation and to the stimulation of downstream signaling pathways 24. Therefore, exploring the regulatory impact of EGFR signaling is of great importance for better understanding the molecular mechanism underlying the pathogenesis of LIHC.

In our study, we provide evidence supporting the oncogenic role of ALYREF-mediated RNA m5C modification in human LIHC. Specifically, ALYREF binds to the m5C-modified EGFR mRNA, enhancing its stability and therefore contributing to the tumorigenesis of LIHC cancer cells. We discovered the association between ALYREF and EGFR in LIHC for the first time and the critical role of ALYREF in controlling the stability of EGFR mRNA in LIHC. The results may help develop a new therapeutic strategy to potentiate ALYREF-targeted EGFR in patients with LIHC.

## Materials and Methods

### Clinical specimens

There are three LIHC cohorts were included in this study. LIHC Cohort 1 (27-paired fresh LIHC tissues and para-cancer normal liver tissues) were collected from the Department of Liver Surgery and Biliary-Pancreatic Surgery at Renji hospital affiliated to Shanghai Jiao Tong University School of Medicine between 2016 and 2018. LIHC Cohort 2 were come from Shanghai Outdo Biotech Ltd (90-paired FFPE LIHC tissues and para-cancer normal liver tissues). LIHC Cohort 3 (152-paired FFPE LIHC tissues and para-cancer normal liver tissues) were collected from the Department of Pathology at Shanghai Ninth People's Hospital affiliated to Shanghai Jiao Tong University School of Medicine between 2008 and 2018. All patients underwent liver resection with no neoadjuvant therapies. RNA (27 pairs) and proteins (7 pairs) were isolated from LIHC Cohort 1 for quantitative real-time PCR (qRT-PCR) and western blotting assay to assess the expression of ALYREF in LIHC. The definition of overall survival (OS) is the interval between the dates of surgery and last follow up or death in LIHC Cohort 2 & 3. In Supplementary Table 1, the clinical pathological characteristics of every LIHC patient are displayed. This study was approved by the Ethical Committee of the Shanghai Ninth People's Hospital and Renji hospital, School of Medicine, Shanghai Jiao Tong University. Before being enrolled, written informed consent was given to each LIHC subject.

### Bioinformatics analysis

LIHC tissues and corresponding prognostic data were downloaded from The Cancer Genome Atlas (TCGA) (https://portal.gdc.cancer.gov/) and were used to analyze the differential expression of m5c-related genes. We retrieved the expression profile data for 26 m5C-related genes from the TCGA database for liver hepatocellular carcinoma (LIHC) samples (n=371) and normal liver tissue samples (n=50). We calculated their respective Z-scores and constructed a heat map using Prism Graphpad 9.0 software. The datasets of GSE62041, GSE214846, GSE77314, GSE13485, GSE94660, GSE45436, GSE136247, and GSE133622 were downloaded from the public source GEO data repository (http://www.ncbi.nlm.nih.gov/geo/). ALYREF binding sites and EGFR m5C sites were obtained by analyzing the datasets of GSE133620 and GSE133622 separately. The Human Protein Atlas (HPA) database (https://www.proteinatlas.org) was used to analyze the expression of ALYREF in LIHC tissues and in the corresponding normal liver tissues.

### Cell lines and culture

Human LIHC cell lines (HepG2, Huh7, Hep3B, Li7), normal human hepatocyte cells (LO2), and HEK293T were obtained from the Shanghai Institute of Biochemistry and Cell Biology, Chinese Academy of Sciences (Shanghai, China) and cultured in RPMI-1640 and DMEM (Gibco, USA). All culture medium was supplemented with 10% fetal bovine serum (FBS) (Gibco, USA), penicillin-streptomycin solution (Gibco, USA), and cultures were maintained in a cell incubator containing 5% CO_2_ at 37 °C.

### Plasmid construction and cell transfection

LIHC cell lines that stably overexpressed ALYREF were constructed using PCDH-CMV-EF1A-T2A-PURO. The PLKO.1-puro vector (Sigma) was used to construct shRNAs targeting ALYREF and the empty vector was used as a negative control. Stable cell lines were obtained after selection with 2 μg/mL puromycin (Beyotime, China) for two weeks. The efficiency of ALYREF overexpression and knockdown was evaluated by qRT-PCR assays and western blotting. The pGL4 luciferase expression vector was used to construct the luciferase reporter plasmid consisting of both renilla luciferase and firefly luciferase. The plasmids were transfected into LIHC cells using Lipofectamine 2000 (Invitrogen) according to the manufacturer's instructions. All the sequences of shRNAs and siRNAs are shown in Supplementary Table 2.

### RNA extraction and qRT-PCR assays

Total RNA was extracted from the cells using Trizol reagent (Invitrogen, USA) and cDNA was synthesized using the PrimerScript RT Reagent Kit (RR037A, Takara Bio, Beijing, China). Gene expression was analyzed using qRT-PCR assays. All primers were purchased from BioSune (Shanghai, China). Relative RNA expression was analyzed using the 2^-ΔΔCt^ method using β-actin for normalization. The primer sequences are shown in Supplementary Table 3.

### Western blotting and antibodies

RIPA lysis buffer (Sangon Biotech) was used for protein extraction. Proteins from cell lysates were separated by sodium dodecyl sulfate (SDS) polyacrylamide gel electrophoresis (6-20%) and then transferred to PVDF membranes. The membranes were blocked with 5% non-fat milk for 2 h at room temperature and incubated with primary antibodies overnight at 4°C. The membranes were then incubated with horseradish peroxidase conjugated secondary antibodies for 1 h at room temperature. The chemiluminescent signals were detected using ECL reagents. Primary antibodies used in this study: anti- Aly/Ref (ab202894) were from abcam, anti-EGFR (4267), anti-STAT3 (9139), anti-p-STAT3 (9145), anti-ERK (4695), anti-p-ERK (8544), anti-AKT (4691S), and anti-p-AKT (4060) were from Cell Signaling Technology (CST). Anti-N-cadherin (A3045) and anti-Snail (A4794) were from Abclonal, while anti-E-cadherin (sc-8426), anti-Slug (sc-166476), and anti-Vimentin (sc-6260) were from Santa Cruz Biotechnology (SCBT).

### Cell proliferation assay

CCK-8 and colony formation assay were performed to measure cell proliferation capacity. For CCK-8 assays, 3000 cells were seeded in each well of 96-well plates and cultured for 72 h. 10 μL volume of CCK-8 solution (Keygen Biotech) was added to each well at 0, 24, 48, and 72 h, and incubated at 37°C for 2 h. The absorbance was detected at an optical density of 450 nm by a microplate reader (BioTek, USA) on the indicated days. Regarding the colony formation assay, 1000 cells per well were digested and cultured in 6-well plates for 14 days. Subsequently, cells were washed with PBS, fixed with 4% paraformaldehyde, and then stained with 1% crystal violet for 20 min, respectively. The number of cell colonies counted and photographed.

### Cell migration assay and invasion assay

Cell migration ability was assessed by the wound-healing assay. Cells layers were scratched forming a straight line using a standard 200 μL pipette tip after cells were cultured to 95% confluence. The scratched cells were incubated with serum-free medium, and the wound area was measured at 0 h and 24 h under an inverted microscope. The wound area was captured and evaluated using ImageJ. Cell invasion ability was assessed using the Transwell invasion assay. 200 μL serum-free medium was loaded into the upper chambers (Corning, USA) and 600 μL medium with 10% FBS was added to the lower compartment. Next, 5×10^4^ cells (Huh7) or 1×10^5^ cells (HepG2) were resuspended with serum-free medium and seeded in the upper chamber coated with Matrigel (BD Biosciences, USA). After 72 h of incubation, the cells were fixed and stained with crystal violet staining solution. The cells were then photographed with a microscope and the number of cells was counted using ImageJ.

### Luciferase reporter assay

For luciferase reporter assays, the EGFR 3' UTR sequence (±500 bp from the predicted m5C site) encoding the wild-type binding site (CCCCC) of ALYREF and corresponding oligos with a mutated binding site (GAGAG) of ALYREF were designed, synthesized, and cloned in the pGL4 promoter vector. The LIHC cells were then seeded in the 24-well plate and transiently transfected with 500 ng of the EGFR 3' UTR luciferase reporter plasmid and 100 ng of the pRL-SV40 Renilla vector using Lipofectamine 2000 (Invitrogen). After cell incubation for 48 h, cells were lysed in passive lysis buffer and the luciferase activity was measured using a dual luciferase assay kit (Beyotime Biotechnology) according to the manufacturer's protocol. The relative luciferase activity was calculated by normalizing the activity of firefly luciferase to that of renilla luciferase.

### Measurement of EGFR mRNA stability

To analyze the stability of EGFR mRNA, stable cells were incubated with 5 μg/mL actinomycin D and collected 0, 2, 4, 6, and 8 h after treatment. Total RNA was extracted and the half-time of the remaining EGFR mRNA was analyzed by qRT-PCR as previously described 25.

### Immunohistochemistry (IHC) staining

In brief, the paraffin sections were deparaffined and treated in methanol with 3% H_2_O_2_ for 20 min. After autoclaving in 10 mM citric sodium (pH 6.0) for 30 min for heat-induced antigen retrieval, they were rinsed twice with PBS and incubated with goat serum. Sections are incubated with primary antibodies at 4°C overnight and then incubated with biotinylated secondary antibody for 1 hour at room temperature. The images were taken with an Olympus camera. ALYREF (HAP019799), EGFR (SAB5700993), Slug (SAB5700672), Snail (SAB5700796), E-cadherin (SAB4503751), N-cadherin (SAB5700641), and Ki67 (ZRB1007) antibodies were obtained from Sigma-Aldrich. Quantification of immunohistochemistry (IHC) staining was based on the positive staining cell rate (ALYREF and Ki67) or IHC score (EGFR, Slug, Snail, E-cadherin, and N-cadherin). The IHC score was calculated from the intensity of the staining (I score: negative, 0; weak, 1; moderate, 2; and intense, 3) and the percentage of positive stained cells (P score: 0-5%, score of 0; 6-35%, score of 1; 36-70%, score of 2; and > 70%, score of 3) to obtain a final score (Q score = I score × P score). Scoring was conducted blindly and independently by two senior pathologists.

### RNA immunoprecipitation (RIP) assay

The RIP assay was performed using the Magna RIP Kit (Millipore, Germany) according to the manufacturer's illustrations. A total of 2 × 10^7^ LIHC cells were lysed in ice-cold RIP buffer. To prepare the magnetic beads, 6 μg anti-EGFR or anti-IgG were coated into the 50 μL protein A/G magnetic beads. For each reaction, 880 μL wash buffer mixed with 35 μL EDTA and 5 μL RNase inhibitor were prepared to synthetize the immunoprecipitation buffer. Protein A/G beads/anti-ALYREF or anti-IgG complexes were then added to this buffer. The RNA of each immunoprecipitate was then purified after washing five times with lysis buffer and digested with proteinase K. Finally, EGFR mRNA levels were analyzed by qRT-PCR.

### Xenografted tumor model and *in vivo* drug studies

A subcutaneous xenograft mouse model was established to assess tumor formation ability. HepG2 cells with ALYREF knockdown, HepG2 cells with ALYREF overexpression, and negative control HepG2 cells (5 × 10^6^) were subcutaneously injected into the flanks of 4-week-old male BALB/c nude mice (six in each group). Tumor volumes and tumor weights were measured every 7 days. All mice were sacrificed, and the tumors were then resected and collected for IHC assays.

For *in vivo* drug studies, HepG2 cells with ALYREF overexpression (5 x 10^6^) or control cells (5 × 10^6^) were subcutaneously injected into the flanks of 4-week-old male BALB/c nude mice (six in each group). When the tumor volume reached a visible tumor size (1 W), mice were randomly assigned to the indicated groups and subjected to treatment with DMSO or erlotinib (40 mg/kg/d) orally. Tumor growth was measured every 7 days. All mice were euthanized, and the tumors were then resected and collected for IHC analysis. All animal studies adhered strictly to the guidelines of the Animal Care and Use Committee of the Shanghai Ninth People's Hospital.

### Statistical analysis

All graphs and analyses were performed using GraphPad Prism 9.0 software. Values are expressed as the Mean ± SD. The student's t-test was used to analyze differences between two groups, and the ANOVA test was used to analyze differences among multiple groups. Receiver operating characteristic (ROC) curve analysis was used to assess the sensitivity and specificity of ALYREF levels for LIHC diagnosis. The Kaplan-Meier method with the logrank test (univariate analysis) and Cox proportional hazards regression model (multivariate analysis) were performed to analyze prognostic factors of patients with LIHC. *P < 0.05; **P < 0.01, and ***P < 0.001 was considered statistically significant.

## Results

### The m5C-related gene ALYREF was highly expressed in LIHC

**Figure 1:**
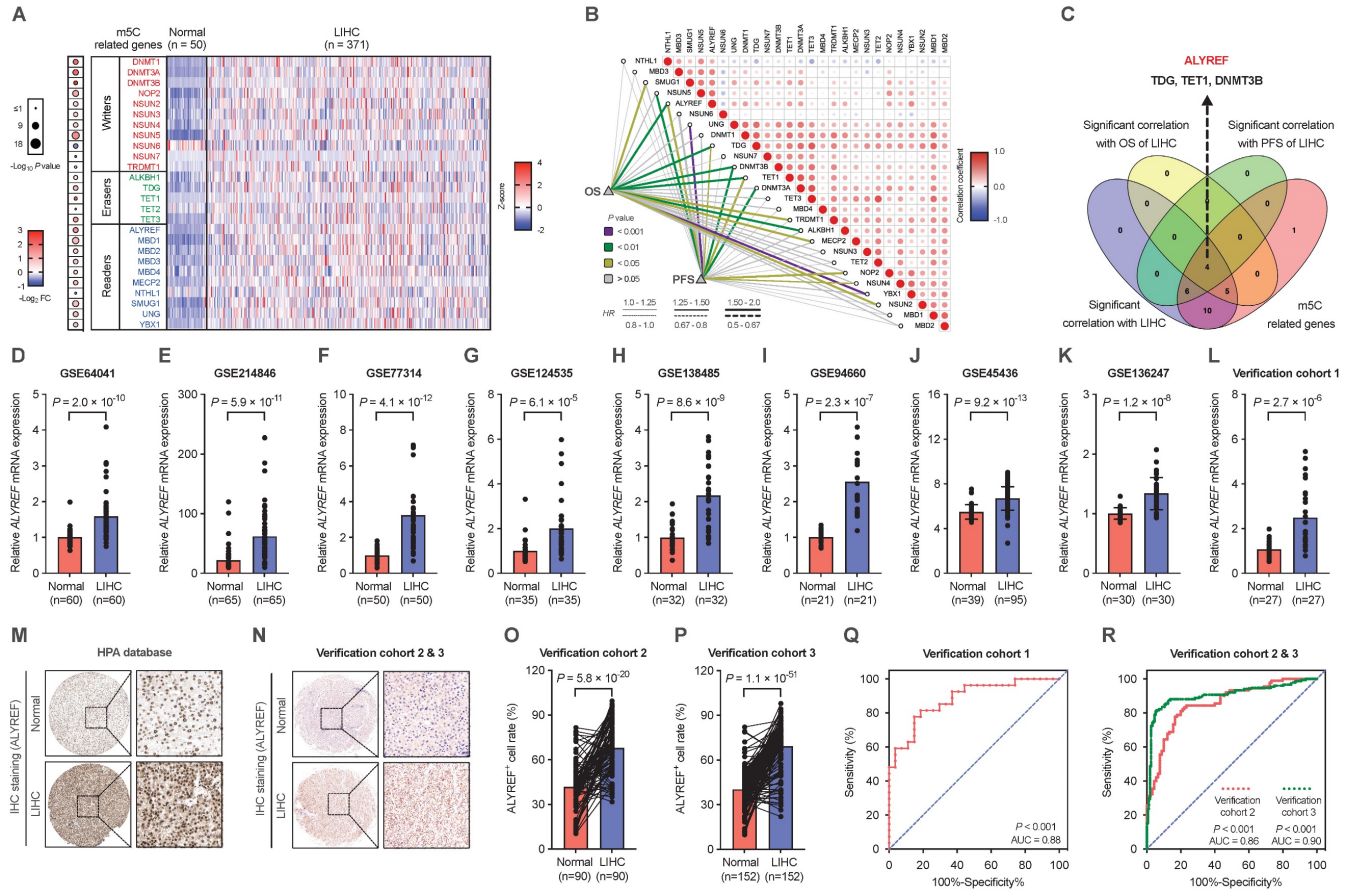
** ALYREF, a m5C-related gene, is highly expressed in LIHC. (A)** Heatmap of the expression of m5C-related genes in LIHC tissues (n = 371) and paired normal tissues (n =50). **(B)** Correlation analysis of m5C-related genes with OS and PFS. **(C)** Venn diagrams showing the expression of overlapping m5C-related genes identified in TCGA databases. **(D-L)** Relative expression of ALYREF mRNA in LIHC samples compared to normal controls analyzed using GEO and in our LIHC cohort 1. **(M, N)** IHC staining of ALYREF in LIHC and normal tissues from HPA databases and our LIHC cohorts 2 and 3. **(O, P)** ALYREF^+^ cell rate (%) in LIHC tumors compared to normal control tissues from our verification cohorts 2 and 3. **(Q, R)** The sensitivity and specificity were shown by the ROC curve for the ALYREF expression in predicting normal with LIHC from cohort 1 (Q) and cohort 2&3 (R). *P < 0.05, **P < 0.01; n.s, not significant; Student's t test.

To determine the possible implications of m5C-related genes in the progression of LIHC, expression of m5C-related genes were analyzed based on the TCGA (https://portal.gdc.cancer.gov/) datasets (Figure 1A). Among them, ALYREF, TDG, TET1, and DNMT3B were significantly correlated with overall survival (OS) and progression-free survival (PFS) of patients with LIHC (Figure 1B,C). Recently, studies have highlighted the roles of TDG, TET1, and DNMT3B in the occurrence and development of LIHC 26-28. Some studies have found that expression of ALYREF is related to the prognosis of LIHC patients and participated in the immune regulation of LIHC. However, the precise correlations between ALYREF-induced RNA m5C modification and LIHC progression remains largely unknown. To verify the promoting effect of ALYREF on LIHC, we examined ALYREF expression in LIHC tissues and their corresponding para-cancer normal tissues using GEO datasets, HPA datasets, and our identification LIHC cohorts (Cohort 1, 2, and 3). Compared to normal liver tissues, both ALYREY mRNA and protein levels were markedly elevated in LIHC tissues (Figure 1D-P). We then analyzed the protein levels of ALYREF in 7 paired LIHC tissues from LIHC cohort 1, and found that ALYREF was significantly upregulated in LIHC (Figure S1A). We also detected ALYREF expression in 4 human LIHC cell lines and normal human hepatocyte cell (LO2) by western blotting and found that ALYREF expression was higher in LIHC cells compared to LO2 (Figure S1B). Finally, we performed a ROC curve analysis to evaluate ALYREF sensitivity and specificity for the diagnosis of LIHC. Indeed, ALYREF achieved a high AUC value in our 3 LIHC cohorts, TCGA-LIHC cohorts, and 8 GEO-LIHC cohorts, indicating that ALYREF had a high sensitivity and specificity for the diagnosis of LIHC (Figure 1Q, R, Figure S1C).

### Expression of ALYREF in LIHC was strongly associated with malignant characteristics and poor prognosis

**Figure 2:**
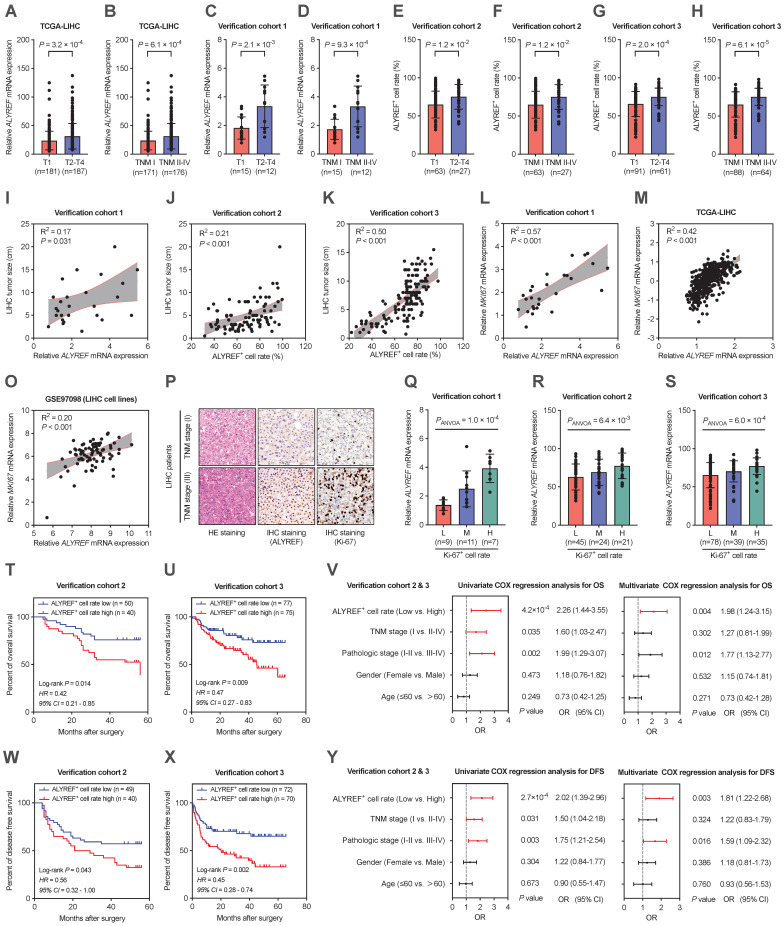
**Correlations between ALYREF and the clinical characteristics of patients with LIHC. (A,B)** ALYREF expression in LIHC samples and normal controls from the TCGA database classified by T1/T2-T4 and TNM I/TNM II-IV. **(C-H)** ALYREF expression in LIHC tissues and normal tissues from our three LIHC cohorts as classified by T1/T2-T4 and TNM I/TNM II-IV. **(I-K)** Correlation analysis between LIHC tumor size and ALYREF expression in our verification cohort 1, 2, and 3. **(L-O)** Correlation analysis between MKI67 and ALYREF mRNA expression from verification cohort 1, TCGA database, and the GEO datasets. **(P)** HE staining, IHC staining (ALYREF), and IHC staining (Ki-67) in stage I TNM, and stage III TNM in LIHC patients. **(Q-S)** Association between the mRNA expression of ALYREF and different levels of Ki-67-postive cell rate from LIHC cohort 1, 2, and 3. **(T, U)** Kaplan-Meier OS curves were assessed to show the overall survival time of LIHC patients from our cohort 2 (**T**) and cohort 3 (**U**). **(V)** Univariableand multivariable COX regression analyses were performed to detect the association between the OS and the clinical parameters in the LIHC patients from cohort 2&3. **(W, X)** Kaplan-Meier DFS curves were assessed to show the disease-free survival time of LIHC patients from our cohort 2 **(W)** and cohort 3 **(X)**. **(Y)** Univariable and multivariable COX regression analyses were performed to detect the association between the DFS and the clinical parameters in the LIHC patients from cohort 2&3. *P < 0.05, **P < 0.01; n.s, not significant; Student's t test.

To explore the clinical importance of ALYREF in LIHC tissues, the relationship between ALYREF levels and several important clinicopathological parameters in the TCGA database and our verification cohorts was evaluated. We found that higher expression of ALYREF mRNA tended to have a more advanced tumor classification and TNM stage both in TCGA-LIHC cohorts and in our 3 LIHC cohorts (Figure 2A-H). Furthermore, both ALYREF mRNA expression and ALYREF^+^ cell rate were closely correlated with tumor size (Figure 2I-K) in LIHC tissues in our verification cohorts. Furthermore, this trend of ALYREF expression was consistent with that of Ki67 expression (Figure 2L-P, Figure S2A-H) and Ki-67^+^ cell rate (Figure. 2Q-S) in the four GEO datasets, TCGA database, and in our 3 LIHC cohorts. We then analyzed the correlation between ALYREF and tumor prognosis using the TCGA database and our 2 LIHC cohorts. The results revealed that patients with higher levels of ALYREF had a poor OS (Figure 2T,U, Figure S2I) and poor DFS (Figure 2W,X, Figure S2K). Furthermore, our univariate COX regression and multivariate COX regression analysis both showed that ALYREF expression was a significant prognostic factor in predicting the OS rate (Figure 2V) and the DFS rate (Figure 2Y) of LIHC patients. Nonetheless, ALYREF expression was found to be an independent correlation factor of OS, but not DFS, in TCGA database (Figure S2J,L). Thus, these results showed that the level of ALYREF might be a potential marker for predicting the prognosis of LIHC patients.

### ALYREF promoted LIHC cell proliferation, migration, and invasion *in vitro* and tumor formation *in vivo*

**Figure 3:**
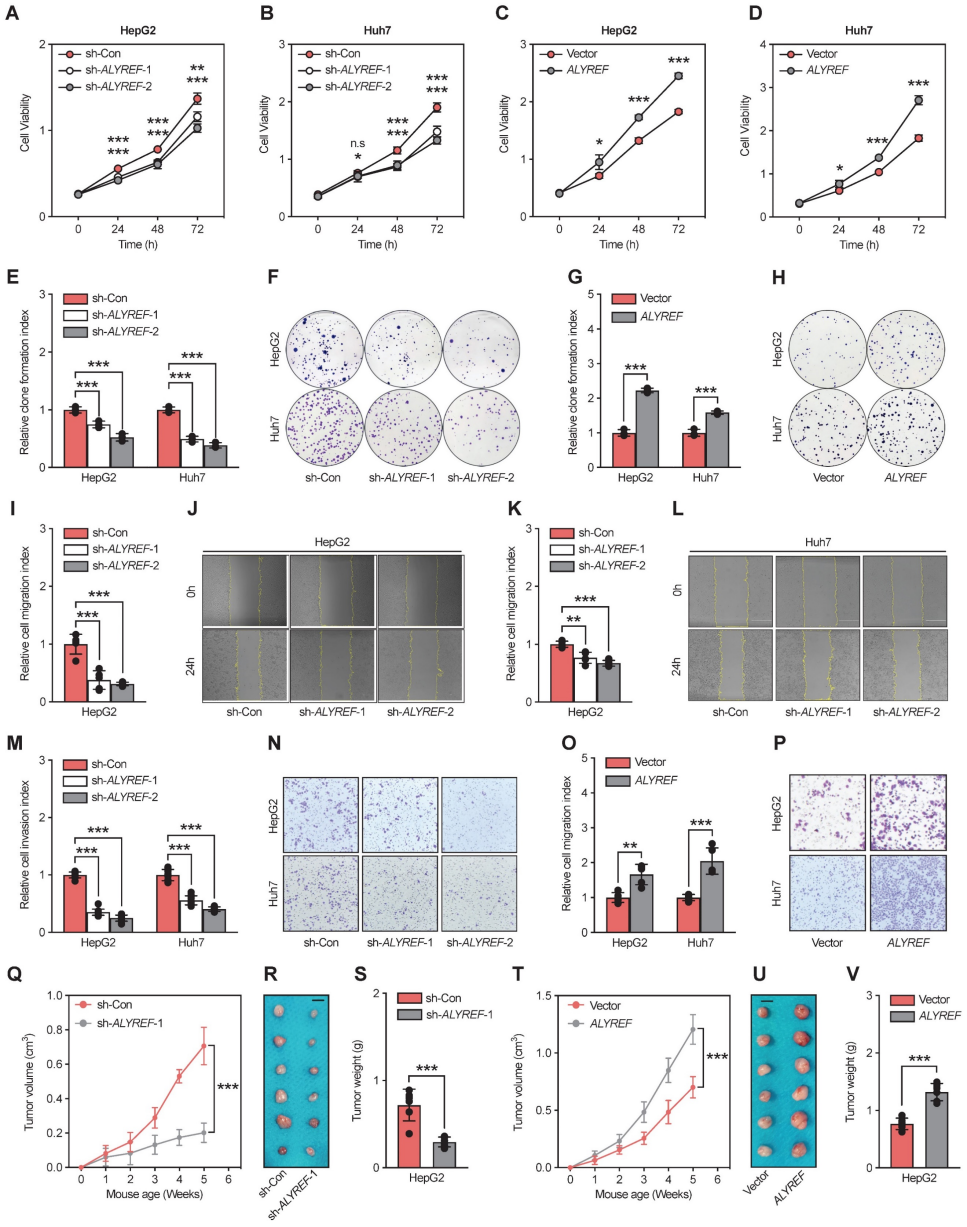
** ALYREF promotes LIHC cell proliferation, migration, and invasion *in vitro* and tumor formation *in vivo*. (A-D)** Cell proliferation was verified by CCK-8 assay. **(E-H)** Representative images and statistical analysis of colony formation assay. **(I-L)** Representative images of the wound healing assays. Scale bar, 200 μm. (M-P) Cell invasion capacity was assessed with Transwell invasion assays. **(Q-S)** Xenograft tumor growth curves, shape, and weight of HepG2 cells transfected with ALYREF shRNA or control shRNA. **(T-V)** Growth curves of tumors from xenografts, shape, and weight of HepG2 cells transfected with the ALYREF overexpression vector or the control vector. *P < 0.05, **P < 0.01; n.s, not significant; Student's t test.

Based on the significant correlation between ALYREF expression and LIHC tumor characteristics, we hypothesized that ALYREF plays a functional role in LIHC progression. To demonstrate the potential biological role of ALYREF in LIHC cells, HepG2 and Huh7 cell lines with stable knockdown or overexpression of ALYREF were constructed, and the efficiency was confirmed by qRT-PCR and western blotting (Figure S3A-D). Interestingly, ALYREF knockdown significantly inhibited LIHC cell growth as evaluated using the CCK8 assay (Figure 3A,B). Conversely, opposite effects on cellular proliferation rates were observed in LIHC cells with ALYREF overexpression (Figure 3C,D). Similar results were obtained for proliferation analysis using colony formation assays (Figure 3E-H). In particular, ALYREF knockdown strongly decreased LIHC cell migration and invasion (Figure 3I-N). Notably, ALYREF overexpression substantially promoted LIHC cell migration and invasion (Figure 3O,P, Figure S3E,F). These *in vitro* results support an oncogenic role of ALYREF in LIHC cells.

To further examine the function of ALYREF in tumor formation *in vivo*, we used HepG2 cells to construct a xenograft mouse tumor model. Consistent with the findings *in vitro*, tumors formed with ALYREF-silenced HepG2 cells showed reduced volume and weights compared to their corresponding control cells (Figure 3Q-S). In contrast, tumors formed with ALYREF-overexpressed HepG2 cells showed faster tumor growth rates and larger tumor weights compared to the control group (Figure 3T-V). IHC staining revealed that ALYREF deficiency reduced the Ki67^+^ cell rate (Figure S3G,H), while ALYREF overexpression increased the Ki67^+^ cell rate (Figure S3I,J) in xenograft tumors respectively. Together, the results confirmed that ALYREF promotes proliferation, migration, and invasion of liver cancer cells *in vitro* and tumor formation and proliferation *in vivo*.

### ALYREF regulated EGFR expression and activated the STAT3 pathway

**Figure 4:**
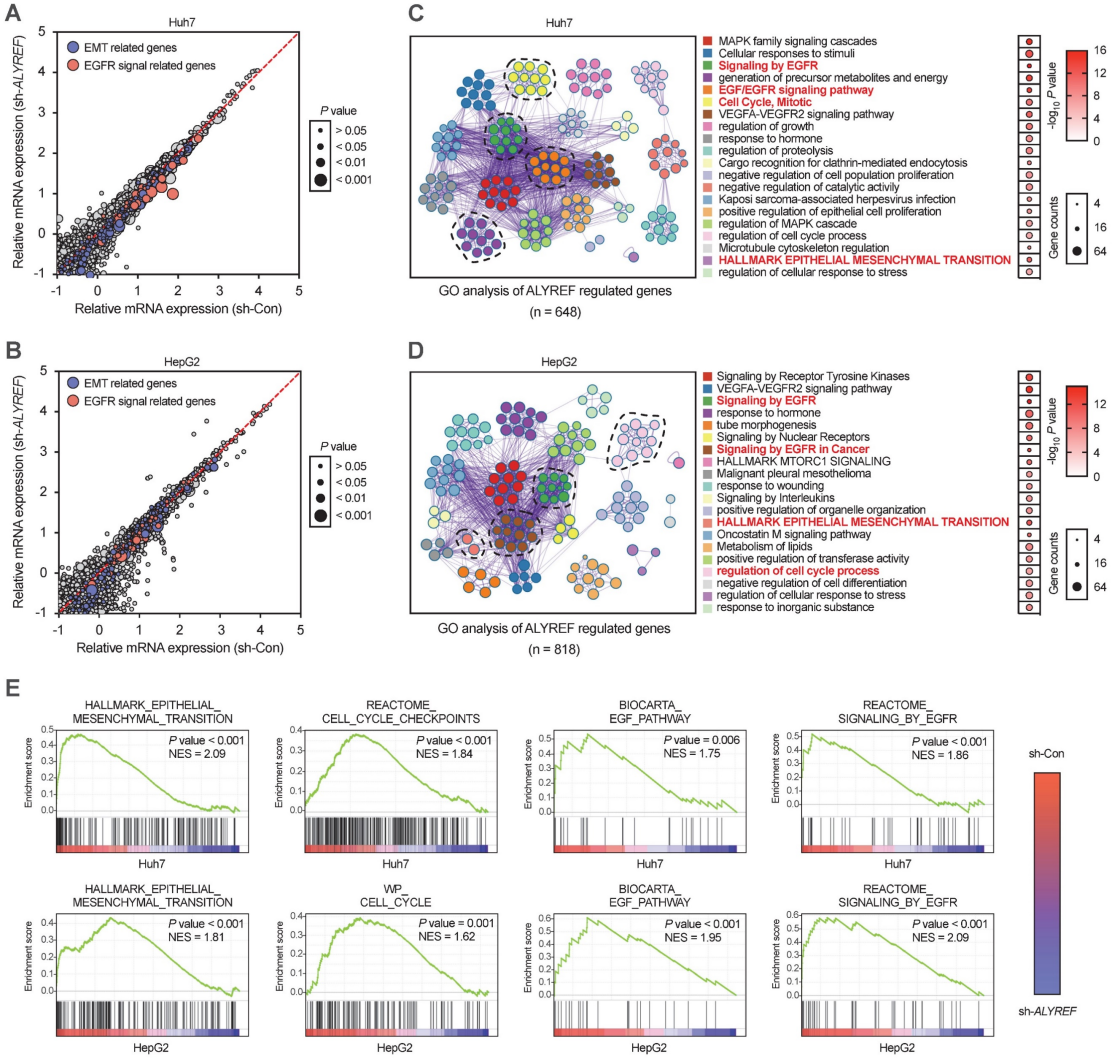
** GO and GSEA analysis of ALYREF-related biological features in LIHC cells transfected with sh-ALYREF or sh-Con. (A, B)** GO analysis of the ALYREF-regulated gene in Huh7 cells and HepG2 cells transfected with sh-ALYREF or sh-Con. **(C, D)** The 20 GO terms with the most significant changes were shown by GO analysis of ALYREF regulated genes. The dot size represents the number of genes detected with decreased expression in ALYREF-knockdown cells compared to control cells associated with that GO term. Dot color represented the P value. GO terms were ranked by their P value. **(E)** GSEA analysis was also performed to investigate the most significant gene sets associated with the ALYREF in LIHC cells. HALLMARK, BIOCARTA, and WP gene sets analysis were performed. *P < 0.05, **P < 0.01; n.s, not significant; Student's t test.

**Figure 5:**
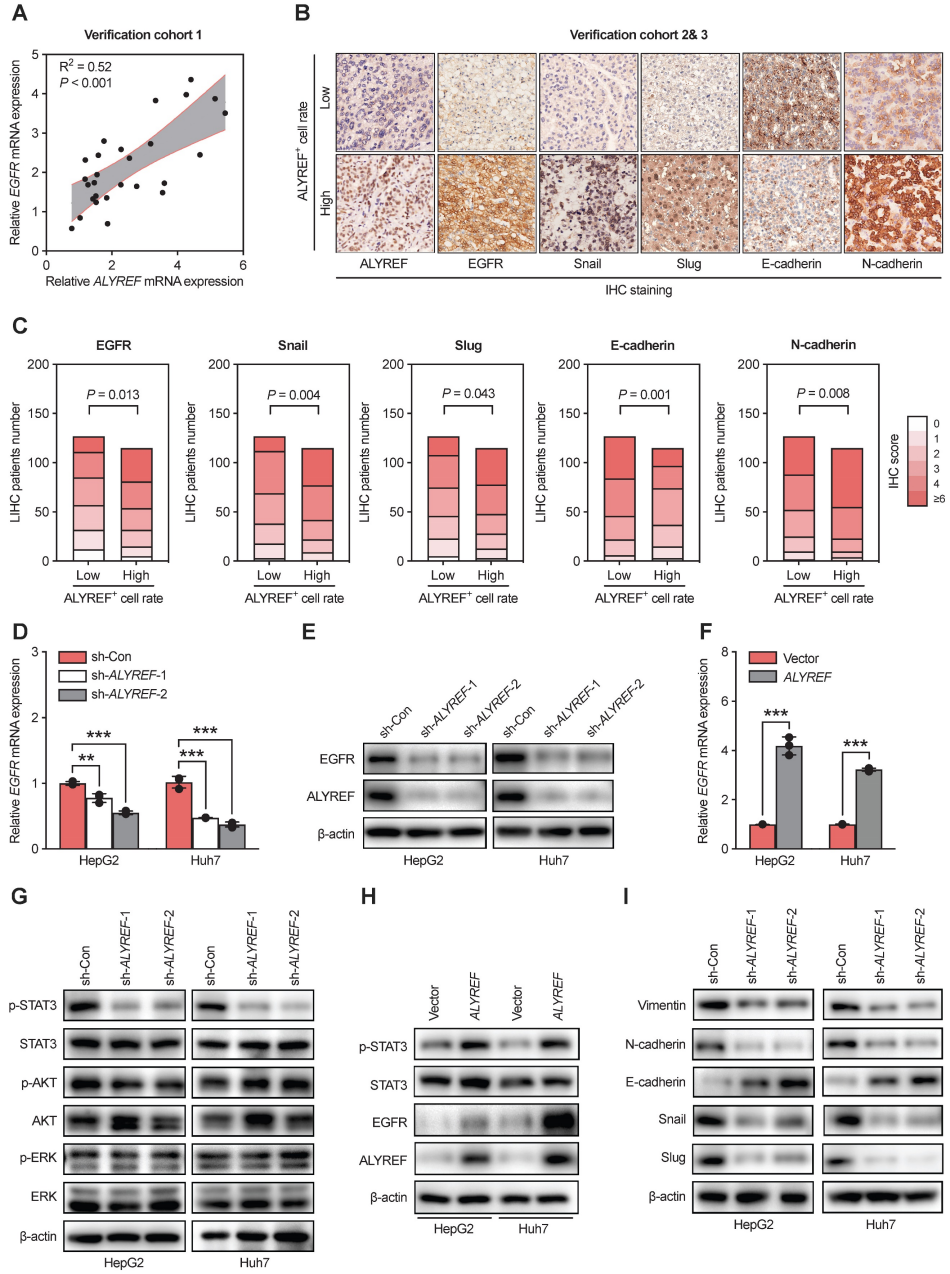
**ALYREF regulates EGFR expression and activates the STAT3 pathway. (A)** Correlation analysis between EGFR and ALYREF mRNA expression in the LIHC verification cohort 1.** (B, C)** IHC staining of EGFR, snail, slug, E-cadherin, N-cadherin in tissues with high ALYREF+ cell signals and tissues with low ALYREF^+^ cell signals in LIHC cohorts 2 and 3. **(D)** EGFR mRNA levels were determined by qRT-PCR assays in LIHC cells with ALYREF knockdown. **(E)** EGFR proteins were measured by western blotting assays in LIHC cells with ALYREF knockdown. **(F)** EGFR mRNA were determined by qRT-PCR assays in LIHC cells overexpressing ALYREF.** (G)** Expression of antibodies targeting components of three main downstream signaling pathways of EGFR as examined by western blotting in LIHC cells with ALYREF knockdown.** (H)** The expression of EGFR, STAT3, and P-STAT3 was examined by western blotting in LIHC cells overexpressing ALYREF. **(I)** The expression of EMT-related proteins after ALYREF knockdown was detected by western blotting assays in LIHC cells. *P < 0.05, **P < 0.01; n.s, not significant; Student's t test.

To further explore the mechanistic basis of the tumor-enhancing effects of ALYREF in LIHC, we performed RNA transcriptome sequencing (RNA-seq) technology to identify the gene expression profiles of LIHC cells with ALYREF knockdown. RNA -seq revealed that ALYREF mRNA expression was significantly correlated with the expression of EMT-related genes and the expression of genes related to EGFR signaling (Figure 4A,B). By using Gene Ontology (GO) enrichment analysis, top 20 downregulated pathways were found (Figure 4C,D). We found that EGFR signaling, cell cycle and EMT processes were correlated with ALYREF expression (Figure 4E). Differentially expressed gene analysis in Huh7 and HepG2 cells (sh-Con vs. sh-ALYREF) revealed that EGFR was significantly down-regulated in LIHC cells harboring ALYREF knockdown (Figure S4). We then confirmed these findings in our verification cohorts. Importantly, there was a positive correlation between EGFR and ALYREF mRNA expression in our LIHC verification cohort 1 (Figure 5A). In LIHC tissues with high-expression ALYREF, IHC staining also revealed an increase in the expression of EGFR (Figure 5B,C), indicating a possible interaction between ALYREF and EGFR. We then performed qRT-PCR and western blotting analyses, which indicated that EGFR was downregulated in LIHC cells with ALYREF knock-down (Figure 5D,E) and was upregulated in LIHC cells overexpressing ALYREF (Figure 5F,H). These results demonstrated that ALYREF could regulate EGFR expression. Three downstream pathways of EGFR have been described to participated in tumorigenesis (i.e., Akt-, Stat3-, and ERK1/2-mediated signaling pathways). Considering the findings described above indicating that ALYREF was correlated with EGFR expression, our objective was to identify the downstream pathways of EGFR that would be inhibited after knockdown of ALYREF expression. Specifically, the response of Akt, STAT3, and ERK1/2 in cells exhibiting ALYREF-silencing was tested and only a decrease in STAT3 phosphorylation was observed, while there were no significant differences in AKT or ERK1/2 phosphorylation (Figure 5G). The activation of p-STAT3 was shown in LIHC cells overexpression of ALYREF (Figure 5H).

EMT is often regarded as a crucial step in the development of LIHC 29. Therefore, we first explored the expression of EMT-related genes through IHC staining in our LIHC cohort 2 and 3 (Figure 5B,C). The expression of epithelial cell marker (E-cadherin) decreased in tissues with high ALYREF^+^ cell rate (Figure 5B,C), while the expression of mesenchymal markers (N-cadherin and vimentin) and EMT-related transcription factors (snail and slug) was increased in tissues with high ALYREF^+^ cell rate (Figure 5B,C). Additionally, knockdown of ALYREF in LIHC cells upregulated the expression of E-cadherin, and decreased the expression of N-cadherin, vimentin, snail and slug (Figure 5I). Similar results were obtained in tumor formation assays (Figure S5A,B).

These results demonstrated that ALYREF could regulate EGFR expression and activate the STAT3 pathway, as well as the EMT processes.

### ALYREF regulated LIHC progression via EGFR signaling

**Figure 6:**
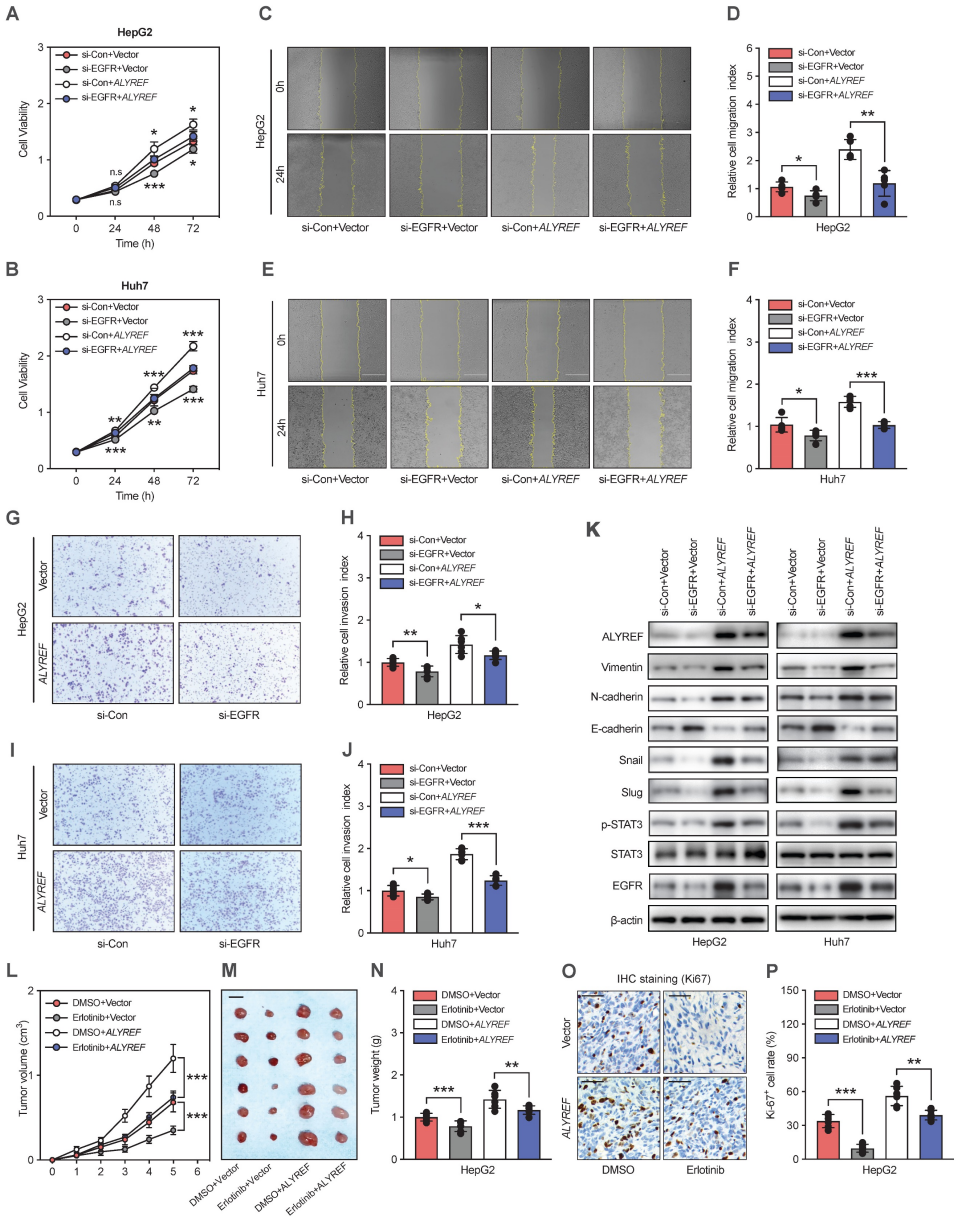
** Silencing of EGFR effectively reverses ALYREF-induced LIHC progression *in vitro* and *in vivo*. (A,B)** CCK8 assays showing the improved cell proliferation capacity resulting from ALYREF overexpression was reversed after inhibiting EGFR expression in LIHC cells. **(C-F)** Wound healing assays showing that the improved migration ability resulting from ALYREF overexpression was reversed after inhibiting EGFR expression in LIHC cells. **(G-J)** Transwell invasion assays showed that the improved invasion ability resulting from ALYREF overexpression was reversed after inhibiting EGFR expression in LIHC cells.** (K)** Western blotting was performed to evaluate the expression of EMT-related proteins, STAT3 and P-STAT3 proteins after transfection with siEGFR in LIHC cells overexpressing ALYREF and control cells. **(L-N)** *In vivo* xenograft tumor formation assays were performed after subcutaneous injection of HepG2 overexpression or control vector cells combined with DMSO or erlotinib treatment (40 mg/kg/day) treatment. The tumor growth curves, shape, and weight of the xenograft were measured. Results are shown as mean ± SEM. **(O)** Representative IHC staining of Ki-67 in tumor xenografts (scale bar: 50 μm). **(P)** The IHC scores of Ki-67 in tumor xenografts. *P < 0.05, **P < 0.01; n.s, not significant; Student's t test.

To further validate whether ALYREF functions in an EGFR signaling-mediated manner in LIHC cells. First, we suppressed the expression of EGFR in ALYREF-overexpressed LIHC cells and control cells using small interference RNA (siEGFR). The CCK-8 assay showed that ALYREF overexpression promoted LIHC cell proliferation, but this was impaired by the silencing of EGFR (Figure 6A-6B). Accordingly, migration and invasion assays revealed that silencing EGFR expression dramatically reversed the migration andinvasion capacity mediated by ALYREF overexpression (Figure 6C-J). Furthermore, western blotting showed that EGFR silencing could partially decreased the expression of N-cadherin, vimentin, snail, and slug promoted by ALYREF overexpression (Figure 6K). However, EGFR silencing could partially restore the expression of E-cadherin which is down-regulated by ALYREF overexpression (Figure 6K). Furthermore, we investigated STAT3 signaling and found that silencing of EGFR led to decreased levels of p-STAT3, which were activated by ALYREF overexpression (Figure 6K).

The EGFR inhibitor erlotinib was used to assess the role of EGFR in ALYREF-induced LIHC progression *in vivo*. Xenograft tumor formation assays in mouse models were performed after subcutaneous injection of HepG2 cells harboring overexpression of ALYREF or control vector HepG2 cells followed by with DMSO or erlotinib treatment. Erlotinib could partially restore the promotion of tumor growth, tumor weight, and Ki-67^+^ cell rate (Figure 6L-P). Furthermore, IHC staining indicated that erlotinib could reverse the effects of ALYREF overexpression on the expression of EMT-related genes (Figure S6A,B). Thus, silencing effectively reversed ALYREF-induced LIHC progression *in vitro* and *in vivo*. Taken together, our results indicated that ALYREF promotes LIHC progression in an EGFR signaling-mediated manner *in vitro* and *in vivo*.

### ALYREF recognized the m5C modification of EGFR and stabilized EGFR mRNA

**Figure 7:**
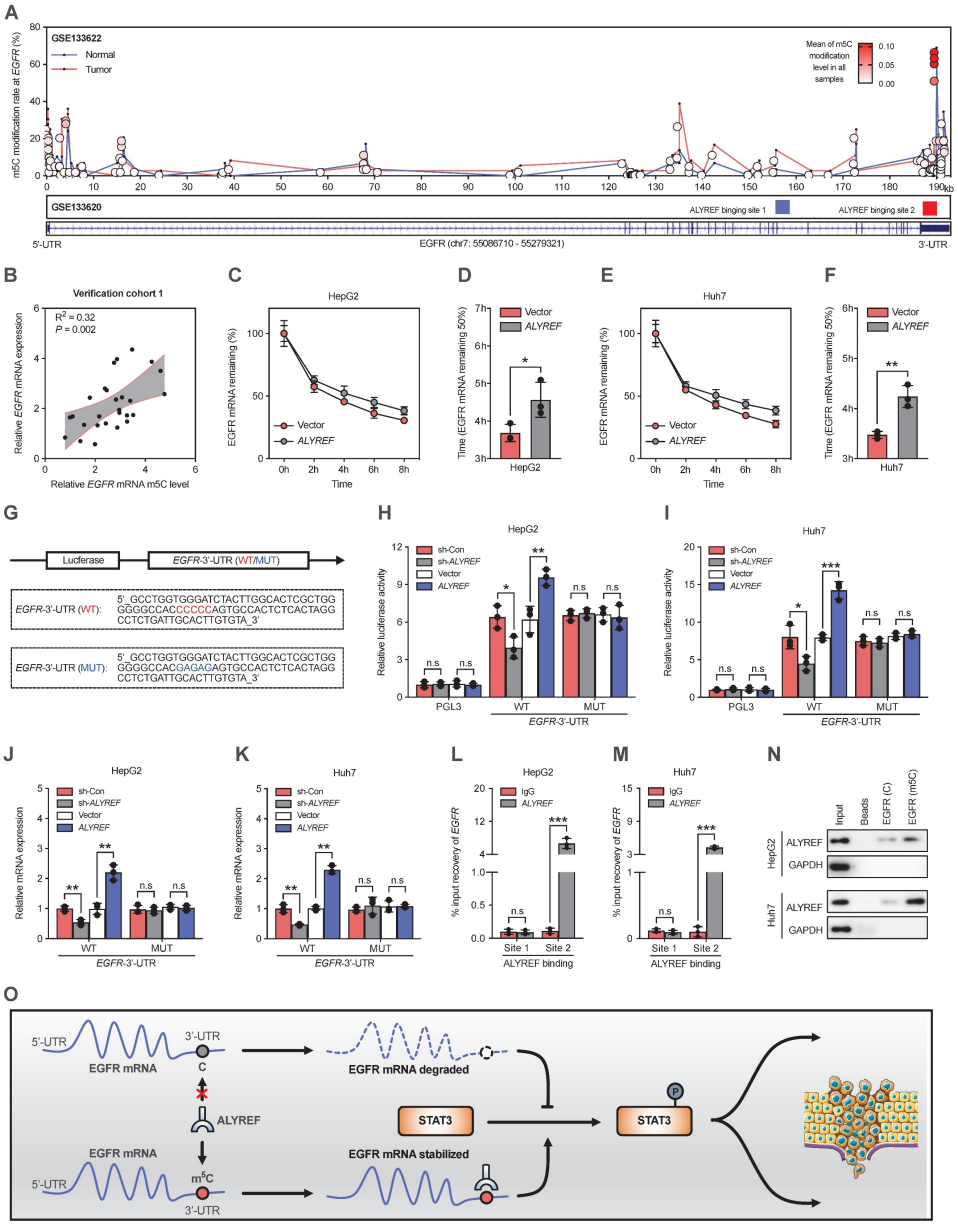
**ALYREF recognizes m5C modification of EGFR and stabilizes EGFR mRNA. (A)** Distribution of m5C peaks and ALYREF-binding peaks across EGFR transcripts by analyzing GSE133622 and GSE133620.** (B)** Correlation between EGFR mRNA and the level of EGFR mRNA m5C level according to the LIHC verification cohort 1. **(C-F)** The indicated stable cells with LIHC were incubated with actinomycin D (5 µg/mL) for 0, 2, 4, 6, and 8 h, and the EGFR mRNA was analyzed by qRT-PCR. **(G)** A schematic drawing showing the m5C binding site on the EGFR 3' UTR (WT) and the corresponding site-specific mutations (MUT). **(H, I)** Relative luciferase activity was measured after stable cells were transfected with the indicated dual-luciferase plasmid including the EGFR m5C site or mutated m5C site.** (J, K)** Relative luciferase mRNA expression was measured after stable LIHC cells were transfected with the indicated dual-luciferase plasmid including the EGFR m5C site or mutant m5C site.** (L-N)** RIP-qPCR validated the interaction between the ALYREF and EGFR m5C modification sites in LIHC cells.** (O)** Model of the mechanisms of action of ALYREF in LIHC. ALYREF may function as an oncogene by stabilizing EGFR mRNA via binding to the m5C site in the EGFR 3' UTR of mRNA, which in turn activates the STAT3 pathway and consequently promotes LIHC progression. *P < 0.05, **P < 0.01; n.s, not significant; Student's t test.

We have proved that ALYREF could upregulate the expression of EGFR at both the mRNA and protein levels in LIHC cells (Figure 5F,5H). Therefore, we hypothesized that ALYREF may closely interact with EGFR. Since the m5C modification is involved in mRNA regulation, we examined the effect of ALYREF m5C modification on EGFR mRNA. First, using integrative ALYREF binding sites and the m5C-modified EGFR sites described in the GSE133620 and GSE133622 datasets separately, we found that the m5C peaks of the EGFR sequence were located in the 3' UTR region (Figure 7A). Importantly, there was a positive correlation between EGFR mRNA and EGFR mRNA m5C level in our LIHC verification cohort 1 (Figure 7B). Furthermore, the mRNA expression of both ALYREF and MKI67 was positively correlated with the level of EGFR mRNA m5C (Figure S7A, S7B). A higher level of EGFR m5C-modified mRNA was associated with advanced T stages and advanced TNM stages in our LIHC verification cohort 1 (Figure S7C,D). These findings suggest there is a correlation between the level of EGFR m5C-modified mRNA and the clinical characteristics of LIHC patients. Consequently, the half-life of EGFR mRNA was significantly increased in LIHC cells that stably expressed ALYREF compared to vector cells after actinomycin D treatment (Figure 7C-F). Using luciferase plasmids encoding the 3ʹ UTR region, we found that luciferase activity and luciferase expression were significantly increased in cells that stably expressed ALYREF compared to vector cells (Figure 7H,I). The increase in luciferase and luciferase expression in cells that stably expressed ALYREF was abrogated in the presence of a mutation of the binding motif (Figure 7G-K). Conversely, opposite effects on luciferase activity were observed in LIHC cells with ALYREF silencing (Figure 7G-K). Furthermore, the RIP-qPCR assay demonstrated that the ALYREF protein directly bound to the EGFR m5C modification sites (Figure 7L-N). Taken together, these results suggest that ALYREF stabilized EGFR mRNA expression by binding the m5C site of EGFR mRNA.

## Discussion

Liver cancer is a common malignancy cancer and is generally aggressive with high mortality rates 30. As a major subtype of liver cancer, LIHC accounts for nearly 80% of all patients with poor prognosis 31. The specific pathophysiological mechanisms that induce the progression of LIHC are not yet clear. Therefore, it is urgent to investigate the underlying molecular mechanisms of LIHC and develop new therapeutic strategies. Dysregulation of epigenetic modifications, including DNA methylation and RNA methylation, have been reported to play a key role in tumor progression. Among these, RNA m5C modification is a common post-transcriptional RNA modification participating in many cellular and physiological processes 32. However, the precise correlations between RNA m5C modification and LIHC progression remains largely unknown.

Accumulating evidence has confirmed that m5C modification regulates RNA stability 33-35, RNA nuclear export 36, and RNA translation 37, 38. RNA m5C modification is catalyzed by several regulaters, among which, ALYREF has attracted increasing interests because of its oncogenic role in various types of cancers 15, 33, 39. By systematically measuring the expression of m5C-related genes related to m5C in the TCGA database, we found that ALYREF as an m5C-related gene exhibited higher expression and significant correlation with OS and PFS in LIHC. It sparked our interests in studying the relevant mechanisms of ALYREF actions on LIHC.

Dysregulation of ALYREF can mediate alterations in the biological function of a variety of tumors. A recent study has found that expression of ALYREF is related to the prognosis of LIHC patients and participated in the immune regulation of LIHC 16, 17, suggesting an important role for LIHC microenvironment. In addition, ALYREF promotes regional lymph node metastasis in oral squamous cell carcinoma and enhances cell proliferation in bladder cancer 15, 40. In our study, we aimed to explore the ALYREF-induced m5C modification on LIHC. By analysing of data from our tissue microarrays and online databases, we found that ALYREF is consistently upregulated in LIHC tissues compared to normal tissues, and upregulated ALYREF is positively correlated with LIHC patients' malignant characteristics and poor prognosis. Thus, these data indicates that ALYREF could be a potential prognostic biomarker. To determine whether the functional activity of ALYREF is relevant for LIHC cellular progression, we performed studies using both ALYREF knockdown cells and ALYREF overexpression cells and found that ALYREF enhanced LIHC proliferation, migration, and invasion *in vitro* and vivo. Our findings suggest that ALYREF is closely related to LIHC cell proliferation and plays a significant biological function in the malignant progression of LIHC.

Upregulation or high activation of EGFR is frequently found in many human cancers. EGFR signaling is an important pathway which is involved in diverse cellular processes, and aberrant EGFR activation occurs in a wide variety of tumors 41. Therefore, targeting the EGFR signaling pathway has been a focus of many drug development efforts in cancer therapy 42. In addition, the critical role of STAT3 as a mediator of the oncogenic effects of EGFR has been demonstrated in several cancers 43. Dysfunction of both pathways is involved in many diseases such as malignant tumors 44. To better detect the molecular mechanism for ALYREF-induced LIHC progression, we first performed mRNA-seq on LIHC cells. Interestingly, we found a positive relationship between ALYREF and EGFR expressiom. Furthermore, we showed that ALYREF stabilized EGFR by binding to the m5C site of EGFR mRNA, which in turn activated the STAT3 signaling pathway and consequently enhanced LIHC progression, suggesting an m5C-dependent oncogenic role of ALYREF towards LIHC.

Our findings have extended the regulatory mechanism of ALYREF expression from an epigenetic perspective and revealed a new mechanism for regulating EGFR expression through m5C modification, indicating that ALYREF-induced m5C epigenetic regulation may have therapeutic benefits in LIHC. Although we have identified ALYREF-targeted m5C binding sites of EGFR mRNA, further investigation is needed to determine whether ALYREF is a common reader and the exact molecular mechanism underlying the association between ALYREF and other m5C-modified mRNAs.

## Conclusions

In this study, we showed that expression of the m5C reader ALYREF was increased in LIHC tissues and its expression was associated with clinicopathological characteristics. The impact of ALYREF on the promotion of LIHC tumorigenicity was further demonstrated *in vitro* and *in vivo*. Additionally, ALYREF functions as an oncogene by stabilizing EGFR expression by binding the m5C site of EGFR mRNA, which in turn activates the STAT3 pathway and promotes LIHC progression. Our study illustrates an ALYREF-induced m5C epigenetic regulatory mechanism of LIHC and highlights the prospect of epitranscriptomic-targeted therapy for LIHC.

## Supplementary Material

Supplementary figures and tables.

## Abbreviations

m5C: 5-Methylcytosine
EMT: Epithelial-mesenchymal transition
FBS: Fetal bovine serum
PVDF: Polyvinylidene fluoride
IHC: Immunohistochemistry
TCGA: The Cancer Genome Atlas
GO: Gene ontology
GSEA: Gene set enrichment analysis
HPA: Human Protein Atlas
RIP: RNA immunoprecipitation assay
Erlotinib: EGFR inhibitor
3'-UTR: 3'-untranslated region
