# Supplementary material for: ALYREF-mediated RNA 5-Methylcytosine modification Promotes Hepatocellular Carcinoma Progression Via Stabilizing EGFR mRNA and pSTAT3 activation
Source: Int J Biol Sci. 2024 Jan 1;20(1):331–46. doi: 10.7150/ijbs.82316 (PMC10750289; doi:10.7150/ijbs.82316)
Supplement: Supplementary file 1 — Supplementary figures and tables. [file ijbsv20p0331s1.pdf]

Supplementary Tables

Supplementary Table 1. Clinical characteristics of 269 LIHC patients from 3 cohorts.

Clinical characteristics	Overall	Verification samples		
		Cohort 1 (n = 27)	Cohort 2 (n = 90)	Cohort 3 (n = 152)
**Follow-up time (OS)**				
Ranges, months	0.7 - 65.5	N.A	5.0 - 56.0	0.7 - 65.5
**Follow-up time (PFS)**				
Ranges, months	0.1 - 65.5	N.A	4.0 - 56.0	0.1 - 65.5
**Gender**				
Mean ± SD, years	57.0 ± 12.4	56.1 ± 12.1	52.4 ± 10.8	59.9 ± 12.6
Female, n (%)	47 (17.5)	4 (14.8)	10 (11.1)	33 (21.7)
Male, n (%)	222 (82.5)	23 (85.2)	80 (88.9)	119 (78.3)
**Age**				
≤ 60 years, n (%)	162 (60.2)	16 (59.3)	71 (78.9)	75 (49.3)
> 60 years, n (%)	107 (39.8)	11 (40.7)	19 (21.1)	77 (50.7)
**Pathological grade**				
I - II, n (%)	163 (60.6)	19 (70.4)	43 (47.8)	101 (66.4)
III - IV, n (%)	106 (39.4)	8 (29.6)	47 (52.2)	51 (33.6)
**Tumor size**				
≤ 5 cm, n (%)	121 (45.0)	11 (40.7)	62 (68.9)	48 (31.6)
> 5 cm, n (%)	148 (55.0)	16 (59.3)	28 (31.1)	104 (68.4)
**T stage**				
T1, n (%)	169 (62.8)	15 (55.6)	63 (70.0)	91 (59.9)
T2-T4, n (%)	100 (37.2)	12 (44.4)	27 (30.0)	61 (40.1)
**N stage**				
N0, n (%)	261 (97.0)	25 (92.6)	90 (100.0)	146 (96.1)
N1, n (%)	8 (3.0)	2 (7.4)	0 (0.0)	6 (3.9)
**M stage**				
M0, n (%)	265 (98.5)	25 (92.6)	90 (100.0)	150 (98.7)
M1, n (%)	4 (1.5)	2 (7.4)	0 (0.0)	2 (1.3)
**TNM stage**				
I, n (%)	165 (61.3)	14 (51.9)	63 (70.0)	88 (57.9)
II - IV, n (%)	104 (38.7)	13 (48.1)	27 (30.0)	64 (42.1)

LIHC, Liver hepatocellular carcinoma; OS, overall survival; DFS, disease-free interval, N.A, not applicable; SD, standard deviation.

Supplementary Table 2. shRNA and siRNA in this study.

	**sequence**
**shALY-1**	CCGGCGTGGAGACAGGTGGGAAACTCTCGAGAGTTTCCCACCTGTCTCCACGTTTTTG
**shALY-2**	CCGGGAACTCTTTGCTGAATTTGGACTCGAGTCCAAATTCAGCAAAGAGTTCTTTTTG
**siEGFR-1**	GUCGCUAUCAAGGAAUUAATT
**siEGFR-2**	CCACAAAGCAGUGAAUUUATT

Supplementary Table 3. Sequences of primers used for qRT-PCR in this study.

	qRT-PCR Primers	
**Gene**	**Forward Sequence(5′-3′)**	**Reverse sequence(5′-3′)**
**ALYREF**	TCTCAGACGCCGATATTCAGG	GTCTGCTGTTCCTAAGCTGCG
**EGFR**	TCCGTGAGTTGATCATCGAATT	TGGAGTCTGTAGGACTTGGCAA
**β-actin**	CATCGGCAATGAGCGGTTC	ACAGCACCGTGTTGGCGTAG

Supplementary Figures

Supplementary Figure 1. (A) Protein expression level of ALYREF in 7- paired LIHC tissues was examined by western blotting. **(B)** Protein expression levels of ALYREF in LO2 and LIHC cell lines. **(C)** The sensitivity and specificity were shown by the ROC curve for the ALYREF expression in predicting normal with LIHC from GEO database.

Supplementary Figure 2. (A-H) Correlation analysis between the MKI67 and ALYREF mRNA expression from the GEO databases. (**I)** Kaplan-Meier OS curves assessed for LIHC patients with high or low ALYREF expression from TCGA databases. (**J)** Univariable and multivariable COX regression analyses were performed to detect the association between the OS and the clinical parameters in the LIHC patients from TCGA databases. (**K)** Kaplan-Meier DFS curves assessed for LIHC patients with high or low ALYREF expression from from TCGA databases. (**L)** Univariable and multivariable COX regression analyses were performed to detect the association between the DFS and the clinical parameters in the LIHC patients from from TCGA databases. Red lines represent ALYREF significantly related clinicopathologic features with $P < 0.05$.

Supplementary Figure 3. (A,B) qPCR analysis and western blotting showing the knockdown efficiency of ALYREF in LIHC cells. **(C,D)** qPCR analysis and western blotting showing the overexpression efficiency of ALYREF in LIHC cells. **(E,F)** Representative images of the wound healing assays for ALYREF overexpression LIHC cells. Scale bar, 200μm. **(G-J)** Representative IHC staining of Ki-67 in tumor xenografts were conducted (scale bar: 50 μm).

Supplementary Figure 4. Heatmap illustrating the differentially expressed genes in LIHC cells between sh-ALYREF and sh-Con groups.

Supplementary Figure 5. (A) IHC staining of EGFR, snail, slug, E-cadherin, N-cadherin in Xenograft tumor transfected with ALYREF shRNA or control shRNA. **(B)** IHC staining of EGFR, snail, slug, E-cadherin, N-cadherin in Xenograft tumor transfected with ALYREF overexpression vector or control vector.

Supplementary Figure 6. (A) Representative IHC staining of EMT-related proteins in tumor xenografts were conducted (scale bar: 50 μm). (B) The IHC staining scores of EMT-related proteins in tumor xenografts.

Supplementary Figure 7. (A) Correlation analysis between the ALYREF mRNA and EGFR mRNA m5C level from LIHC cohort 1. (B) Correlation analysis between the MKI67 mRNA and EGFR mRNA m5C level from LIHC cohort 1. (C) Association between the level of EGFR mRNA m5C and different T stages from LIHC cohort 1. (D) Association between the level of EGFR mRNA m5C and different TNM stages from LIHC cohort 1.

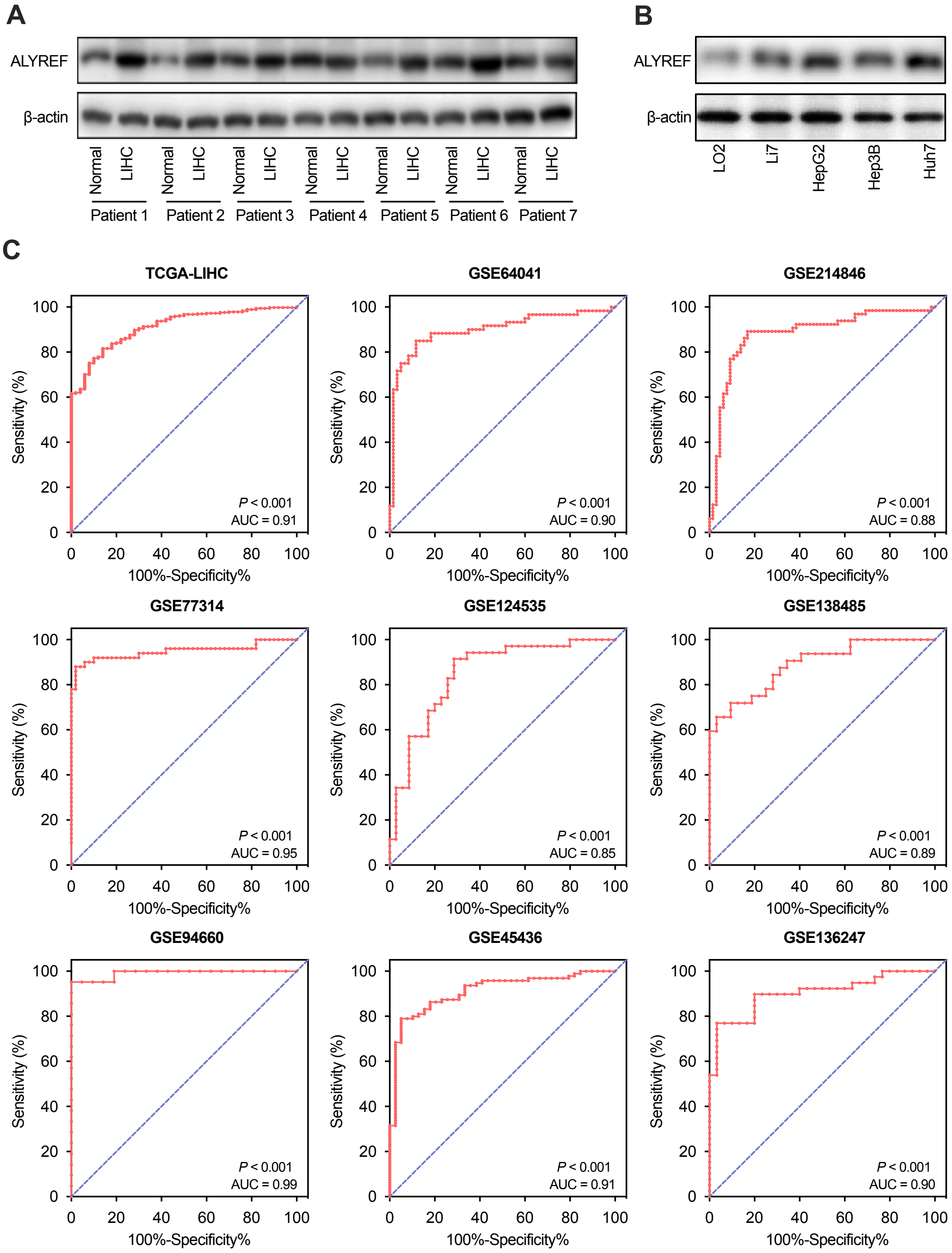
A
ALYREF
β-actin
Normal
LIHC
Patient 1
Patient 2
Patient 3
Patient 4
Patient 5
Patient 6
Patient 7
B
ALYREF
β-actin
LO2
Li7
HepG2
Hep3B
Huh7
C
TCGA-LIHC
P < 0.001
AUC = 0.91
GSE64041
P < 0.001
AUC = 0.90
GSE214846
P < 0.001
AUC = 0.88
GSE77314
P < 0.001
AUC = 0.95
GSE124535
P < 0.001
AUC = 0.85
GSE138485
P < 0.001
AUC = 0.89
GSE94660
P < 0.001
AUC = 0.99
GSE45436
P < 0.001
AUC = 0.91
GSE136247
P < 0.001
AUC = 0.90
Sensitivity (%)
100%-Specificity%

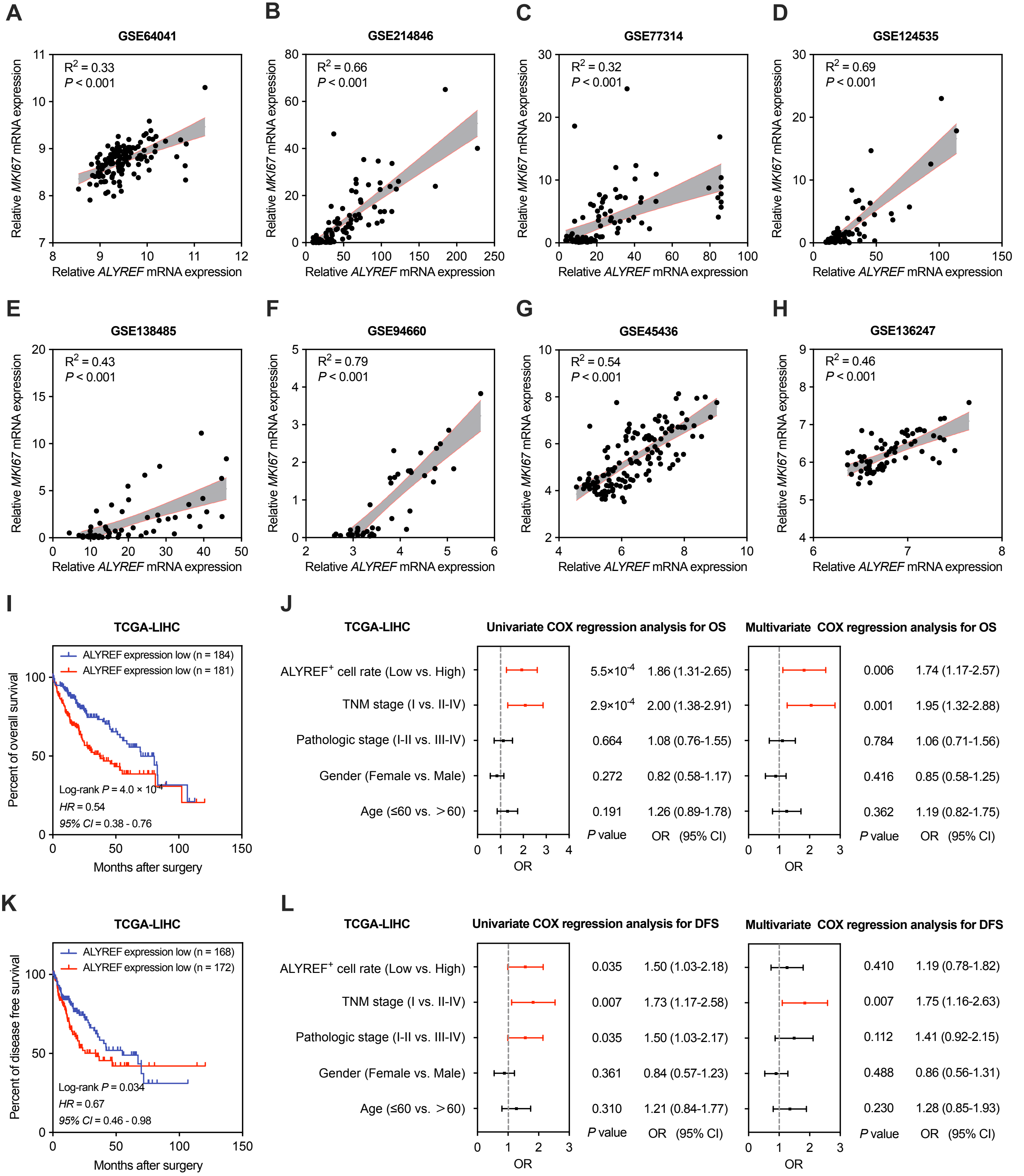

A
GSE64041
R^2 = 0.33
P < 0.001
Relative MK67 mRNA expression
Relative ALYREF mRNA expression
B
GSE214846
R^2 = 0.66
P < 0.001
C
GSE77314
R^2 = 0.32
P < 0.001
D
GSE124535
R^2 = 0.69
P < 0.001
E
GSE138485
R^2 = 0.43
P < 0.001
F
GSE94660
R^2 = 0.79
P < 0.001
G
GSE45436
R^2 = 0.54
P < 0.001
H
GSE136247
R^2 = 0.46
P < 0.001
I
TCGA-LIHC
ALYREF expression low (n = 184)
ALYREF expression low (n = 181)
Percent of overall survival
Log-rank P = 4.0 × 10^{-4}
HR = 0.54
95% CI = 0.38 - 0.76
Months after surgery
J
TCGA-LIHC
Univariate COX regression analysis for OS
Multivariate COX regression analysis for OS
ALYREF$^+$ cell rate (Low vs. High)
TNM stage (I vs. II-IV)
Pathologic stage (I-II vs. III-IV)
Gender (Female vs. Male)
Age (≤60 vs. >60)
5.5×10^{-4} 1.86 (1.31-2.65)
2.9×10^{-4} 2.00 (1.38-2.91)
0.664 1.08 (0.76-1.55)
0.272 0.82 (0.58-1.17)
0.191 1.26 (0.89-1.78)
0.006 1.74 (1.17-2.57)
0.001 1.95 (1.32-2.88)
0.784 1.06 (0.71-1.56)
0.416 0.85 (0.58-1.25)
0.362 1.19 (0.82-1.75)
P value
OR (95% CI)
OR
K
TCGA-LIHC
ALYREF expression low (n = 168)
ALYREF expression low (n = 172)
Percent of disease free survival
Log-rank P = 0.034
HR = 0.67
95% CI = 0.46 - 0.98
Months after surgery
L
TCGA-LIHC
Univariate COX regression analysis for DFS
Multivariate COX regression analysis for DFS
ALYREF$^+$ cell rate (Low vs. High)
TNM stage (I vs. II-IV)
Pathologic stage (I-II vs. III-IV)
Gender (Female vs. Male)
Age (≤60 vs. >60)
0.035 1.50 (1.03-2.18)
0.007 1.73 (1.17-2.58)
0.035 1.50 (1.03-2.17)
0.361 0.84 (0.57-1.23)
0.310 1.21 (0.84-1.77)
0.410 1.19 (0.78-1.82)
0.007 1.75 (1.16-2.63)
0.112 1.41 (0.92-2.15)
0.488 0.86 (0.56-1.31)
0.230 1.28 (0.85-1.93)
P value
OR (95% CI)
OR

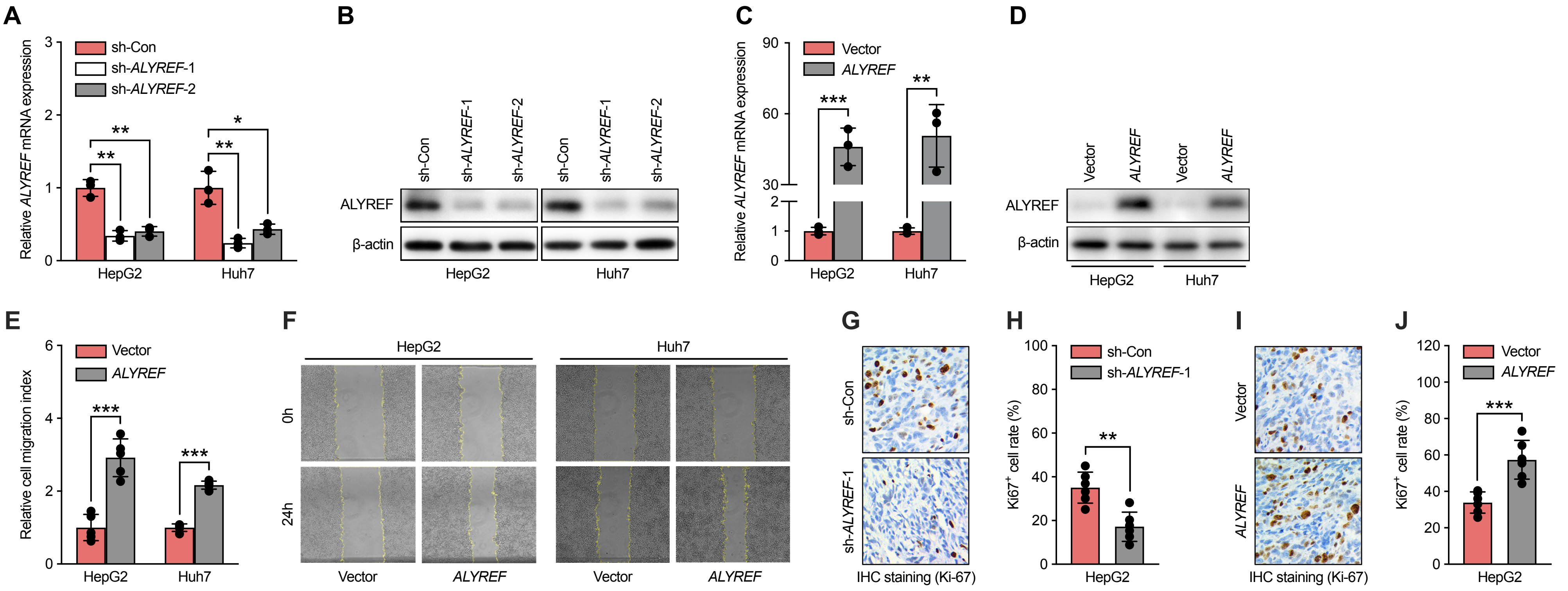
A
Relative ALYREF mRNA expression
sh-Con
sh-ALYREF-1
sh-ALYREF-2
HepG2
Huh7
B
sh-Con
sh-ALYREF-1
sh-ALYREF-2
ALYREF
β-actin
HepG2
Huh7
C
Relative ALYREF mRNA expression
Vector
ALYREF
HepG2
Huh7
D
Vector
ALYREF
ALYREF
β-actin
HepG2
Huh7
E
Relative cell migration index
Vector
ALYREF
HepG2
Huh7
F
HepG2
Huh7
0h
24h
Vector
ALYREF
G
sh-Con
sh-ALYREF-1
IHC staining (Ki-67)
H
Ki67+ cell rate (%)
sh-Con
sh-ALYREF-1
HepG2
I
Vector
ALYREF
IHC staining (Ki-67)
J
Ki67+ cell rate (%)
Vector
ALYREF
HepG2

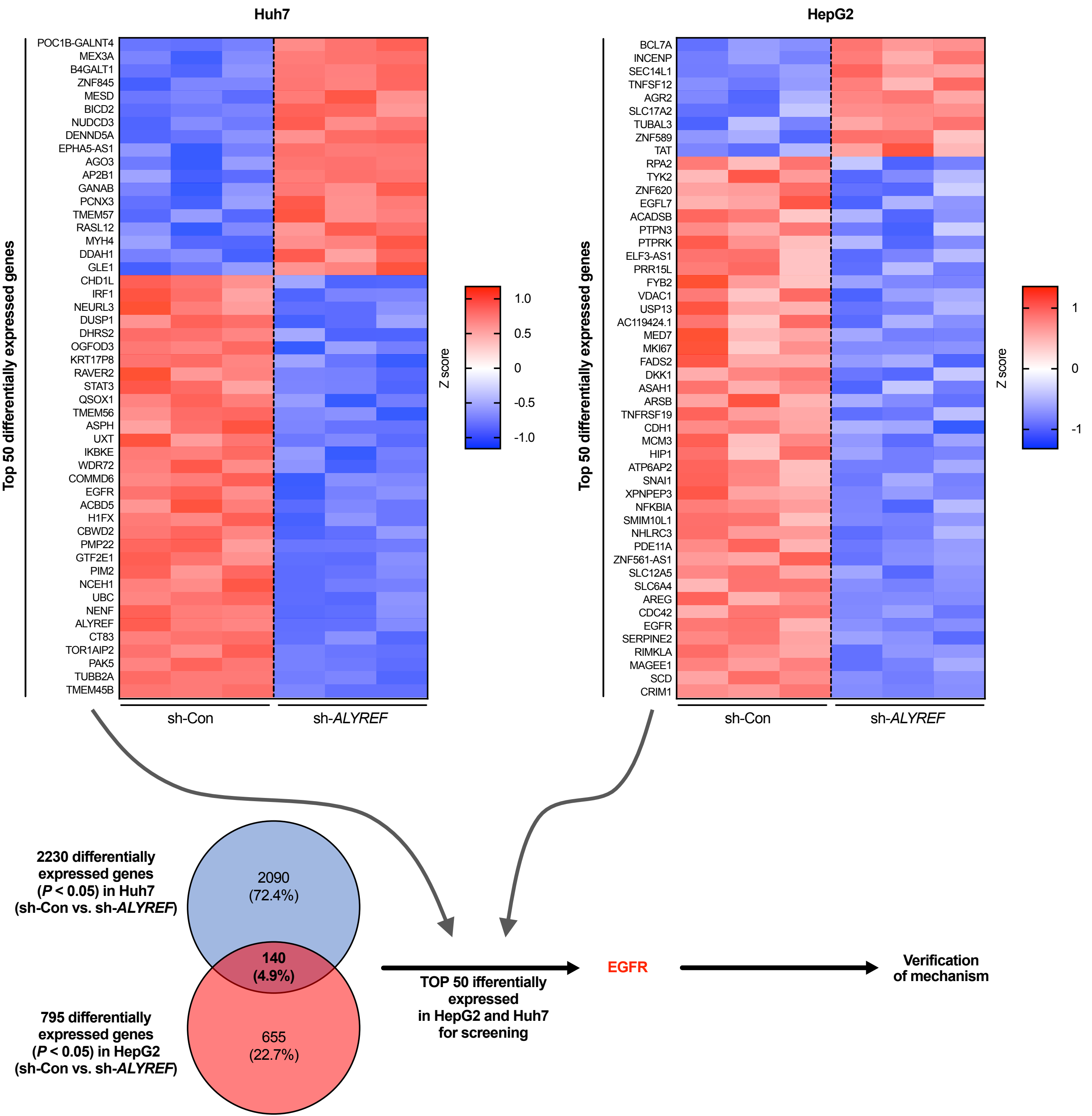
Huh7
HepG2
Top 50 differentially expressed genes
POC1B-GALNT4
MEX3A
B4GALT1
ZNF845
MESD
BICD2
NUDCD3
DENND5A
EPHA5-AS1
AGO3
AP2B1
GANAB
PCNX3
TMEM57
RASL12
MYH4
DDAH1
GLE1
CHD1L
IRF1
NEURL3
DUSP1
DHRS2
OGFOD3
KRT17P8
RAVER2
STAT3
QSOX1
TMEM56
ASPH
UXT
IKBKE
WDR72
COMMD6
EGFR
ACBD5
H1FX
CBWD2
PMP22
GTF2E1
PIM2
NCEH1
UBC
NENF
ALYREF
CT83
TOR1AIP2
PAK5
TUBB2A
TMEM45B
sh-Con
sh-ALYREF
Z score
1.0
0.5
0
-0.5
-1.0
BCL7A
INCENP
SEC14L1
TNFSF12
AGR2
SLC17A2
TUBAL3
ZNF589
TAT
RPA2
TYK2
ZNF620
EGFL7
ACADSB
PTPN3
PTPRK
ELF3-AS1
PRR15L
FYB2
VDAC1
USP13
AC119424.1
MED7
MKI67
FADS2
DKK1
ASAH1
ARSB
TNFRSF19
CDH1
MCM3
HIP1
ATP6AP2
SNAI1
XPNPEP3
NFKBIA
SMIM10L1
NHLRC3
PDE11A
ZNF561-AS1
SLC12A5
SLC6A4
AREG
CDC42
EGFR
SERPINE2
RIMKLA
MAGEE1
SCD
CRIM1
1
0
-1
2230 differentially expressed genes (P < 0.05) in Huh7 (sh-Con vs. sh-ALYREF)
795 differentially expressed genes (P < 0.05) in HepG2 (sh-Con vs. sh-ALYREF)
2090 (72.4%)
140 (4.9%)
655 (22.7%)
TOP 50 ifferentially expressed in HepG2 and Huh7 for screening
EGFR
Verification of mechanism

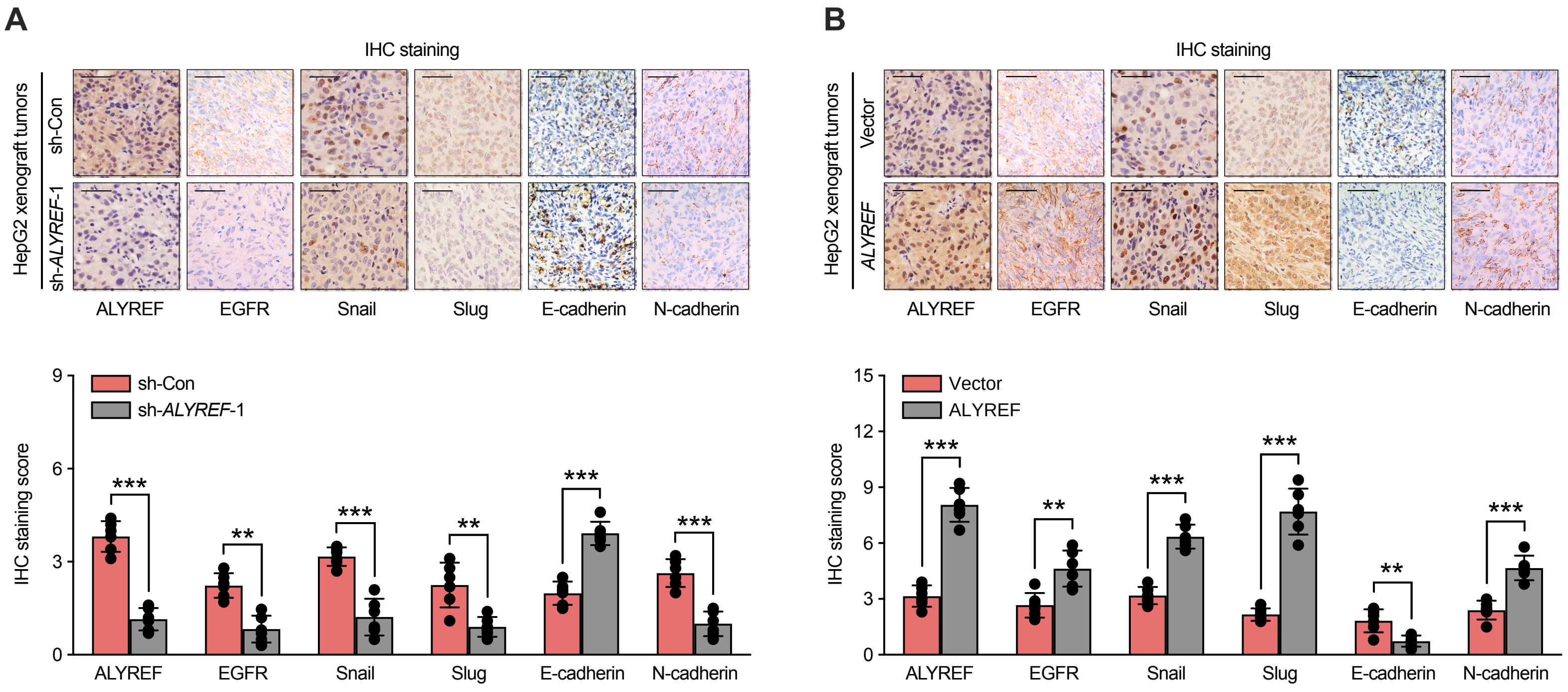
A
IHC staining
HepG2 xenograft tumors
sh-Con
sh-ALYREF-1
ALYREF
EGFR
Snail
Slug
E-cadherin
N-cadherin
IHC staining score
sh-Con
sh-ALYREF-1
B
IHC staining
HepG2 xenograft tumors
Vector
ALYREF
IHC staining score
Vector
ALYREF

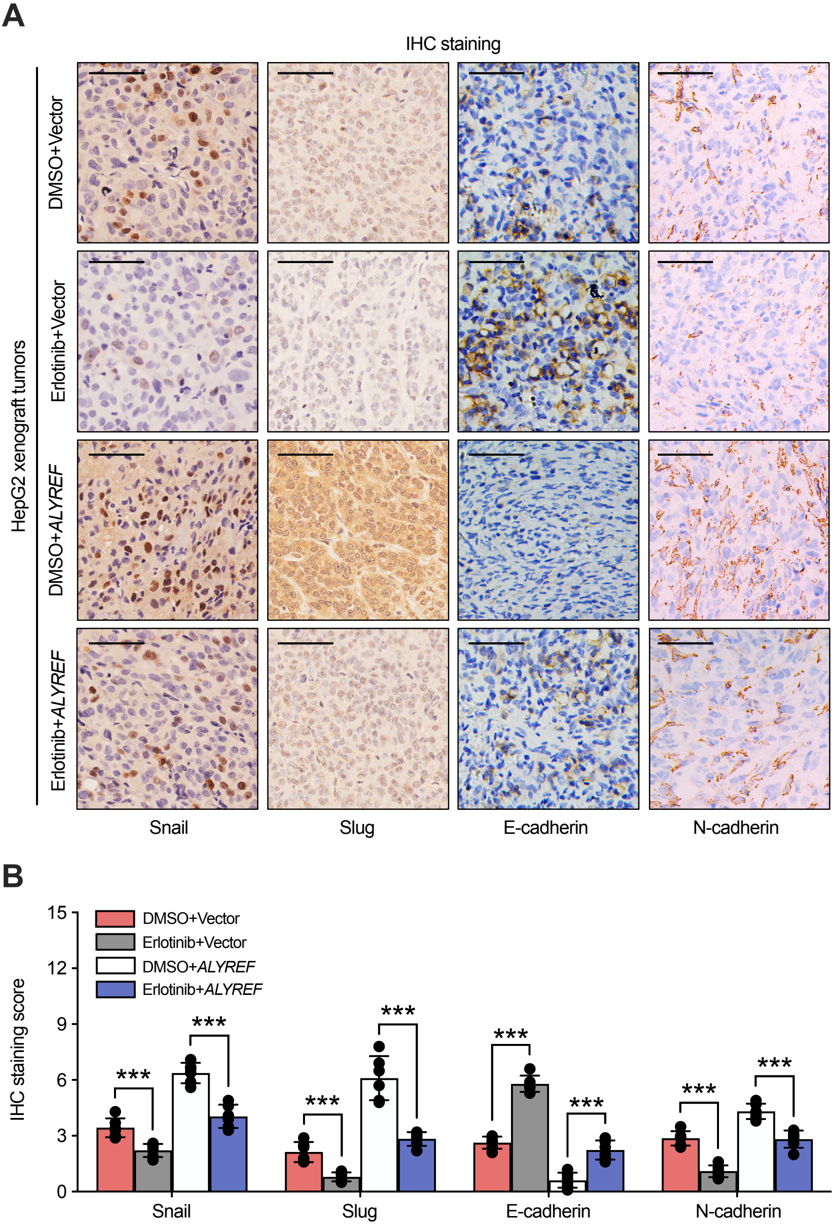
A
IHC staining
HepG2 xenograft tumors
DMSO+Vector
Erlotinib+Vector
DMSO+ALYREF
Erlotinib+ALYREF
Snail
Slug
E-cadherin
N-cadherin
B
IHC staining score
15
12
9
6
3
0
DMSO+Vector
Erlotinib+Vector
DMSO+ALYREF
Erlotinib+ALYREF
***
Snail
Slug
E-cadherin
N-cadherin

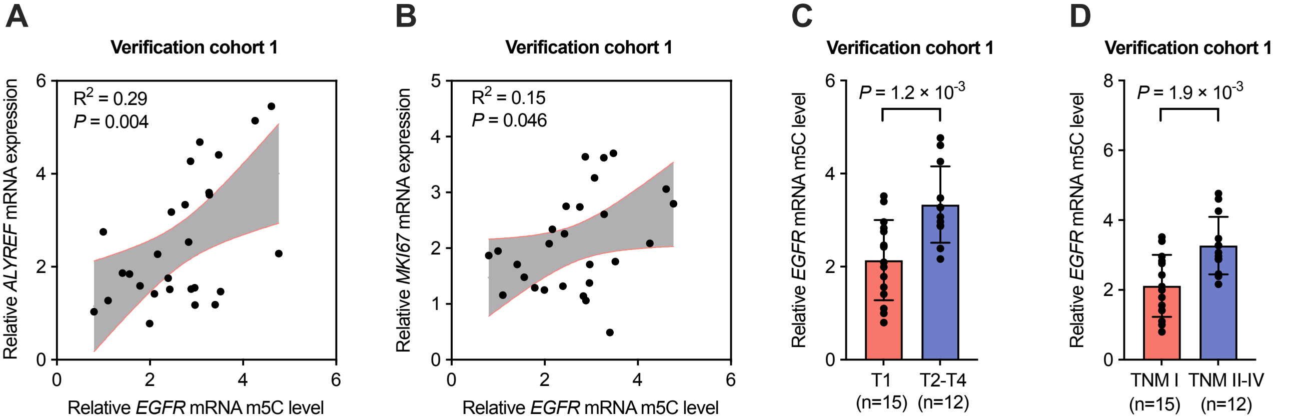
A
Verification cohort 1
R^2 = 0.29
P = 0.004
Relative ALYREF mRNA expression
Relative EGFR mRNA m5C level
B
Verification cohort 1
R^2 = 0.15
P = 0.046
Relative MKI67 mRNA expression
Relative EGFR mRNA m5C level
C
Verification cohort 1
P = 1.2 × 10^{-3}
Relative EGFR mRNA m5C level
T1 (n=15)
T2-T4 (n=12)
D
Verification cohort 1
P = 1.9 × 10^{-3}
Relative EGFR mRNA m5C level
TNM I (n=15)
TNM II-IV (n=12)
